# Technical Feasibility and Preliminary Safety Analysis of Intraoperative Electromyographic Neuromonitoring Using an Insulated Access Needle During Minimally Invasive Lumbar Pedicle Screw Fixation: A Retrospective Multicenter Series

**DOI:** 10.7759/cureus.110071

**Published:** 2026-06-01

**Authors:** Fred Mo, Behnam Myers, Rajesh V Patel, Travis Greenhalgh, Bob Salvat

**Affiliations:** 1 Department of Orthopedic Surgery, MedStar Georgetown University Hospital, Washington, USA; 2 Department of Orthopedic Surgery, Nova Southeastern University, Fort Lauderdale, USA; 3 Department of Orthopedic Surgery, University of Pennsylvania Perelman School of Medicine, Philadelphia, USA; 4 Department of Engineering, SurGenTec, Limited Liability Company, Boca Raton, USA; 5 Department of Clinical Research, SurGenTec, Limited Liability Company, Boca Raton, USA

**Keywords:** insulated access needle, intraoperative neuromonitoring, jamshidi needle, lumbar spine surgery, minimally invasive spine surgery, pedicle breach, pedicle screw fixation, stimulation threshold, transforaminal lumbar interbody fusion (tlif), triggered electromyography

## Abstract

Background

Triggered electromyography (t-EMG) is used as an adjunct to fluoroscopy during minimally invasive lumbar pedicle screw fixation to detect potential cortical breaches. Its diagnostic accuracy depends on the electrical isolation properties of the stimulating instrument. Standard non-insulated Jamshidi needles allow current to disperse along the full needle shaft, which may reduce signal specificity at the pedicle tip. An insulated access needle with an adjustable depth stop (ALARA™ Neuro Access Needle; SurGenTec, LLC, Boca Raton, FL, USA) was used with continuous t-EMG monitoring in this series. Screw position was assessed by biplanar fluoroscopy and final t-EMG testing only; no postoperative CT was obtained in asymptomatic patients.

Methods

Retrospective post-market multi-center series. Three surgeons at independent sites placed 367 percutaneous lumbar pedicle screws in 75 consecutive patients (182 spinal levels, L1-S1, 2020-2021). An ALARA needle threshold of ≥5 mA was the adopted criterion before proceeding to guidewire placement. All patients underwent postoperative neurological assessment.

Results

No patient sustained a new postoperative neurological deficit attributable to screw malposition. Of 367 screws, 341 (92.9%) were placed without needle repositioning; 26 (7.1%) required repositioning because of sub-threshold EMG readings. One screw (0.3%) produced persistently low EMG values (final screw reading, 9 mA at L4) consistent with possible pedicle cortical proximity; this was flagged clinically, and the patient experienced no neurological sequelae on follow-up examination. ALARA needle readings of ≥5 mA preceded final screw readings of ≥10 mA in all cases in which repositioning was not required, an empirical offset consistent with differences in stimulating surface area between the needle tip and the screw.

Conclusions

Use of an insulated access needle with continuous t-EMG monitoring identified and enabled real-time correction of suboptimal needle trajectories in 26 of 367 screws (7.1%) before guidewire placement, with zero postoperative neurological deficits across 75 patients at three independent surgical sites using multiple implant systems. A needle-specific threshold of ≥5 mA consistently preceded final screw readings within the published safety range across all treated lumbar and sacral levels. These findings support needle-stage t-EMG monitoring with an insulated access needle as a practical, platform-independent tool for enhancing intraoperative safety during minimally invasive surgical (MIS) lumbar pedicle screw fixation, with prospective CT-correlated studies warranted to establish definitive threshold standards.

## Introduction

Pedicle screw instrumentation is the standard approach to posterior spinal stabilization across degenerative, deformity, and traumatic pathologies [1,2]. Percutaneous minimally invasive surgical (MIS) techniques have become increasingly prevalent, offering reduced blood loss, diminished paraspinal muscle disruption, and shorter hospitalization compared with open approaches [1]. However, percutaneous access eliminates the direct tactile and visual feedback available during open surgery, placing greater reliance on fluoroscopy and adjunct monitoring to confirm pedicle wall integrity before screw insertion.

Population-level data report pedicle screw malposition rates of 3.7% to 31.5%, depending on technique, spinal level, and assessment methodology [3,4]. Medially directed violations carry the highest clinical consequence, placing the traversing nerve root or thecal sac at risk of injury. Neurological complications from malpositioned screws, including radiculopathy, motor deficit, and cauda equina syndrome, represent serious and potentially irreversible adverse outcomes [5].

Triggered electromyography (t-EMG) is an established neurophysiological adjunct to fluoroscopy for pedicle screw placement [6]. The underlying principle is that electrical stimulation of an instrument positioned within intact pedicle bone requires greater current intensity to activate adjacent motor nerve fibers than if the instrument abuts or penetrates exposed neural tissue. An important aspect of this framework is that safe and unsafe stimulation threshold values are not absolute constants; they depend on the surface area of the stimulating interface and the electrical conductivity of the surrounding medium. Consequently, published threshold norms derived from pedicle screw stimulation studies are not directly applicable to access needle stimulation, which presents a fundamentally different geometry within the same anatomical environment.

Foundational t-EMG threshold data for lumbar pedicle screws established that stimulation values above 15 mA confer a 98% probability of intrapedicular placement, while values below 10 mA suggest cortical perforation [7]. Raynor et al., in an analysis of 4857 lumbar pedicle screws, demonstrated a continuous increase in medial breach probability with decreasing threshold: 0.31% above 8 mA, 17.4% between 4-8 mA, and 54.2% below 4 mA [8]. A meta-analysis of 13948 lumbar screws identified ≤8 mA as the optimal diagnostic cutoff, with a sensitivity of 0.82 and a specificity of 0.97 for malposition [9]. These data apply to pedicle screws specifically; their direct application to access needle stimulation would be methodologically inappropriate without accounting for the geometric difference in stimulating interface.

Standard Jamshidi access needles are constructed of conductive metal without insulation. When stimulating current is applied, it disperses along the full needle shaft into surrounding soft tissue, reducing effective current density at the needle tip and potentially degrading signal specificity [10]. An insulated access needle limits current delivery to the unshielded needle tip, theoretically providing more anatomically localized stimulation. This technical note reports a preliminary multi-center retrospective safety series using one such device, the ALARA Neuro Access Needle Kit (SurGenTec, LLC, Boca Raton, FL, USA; FDA 510(k)-cleared), with the primary aim of documenting the rate of clinically evident neurological injury and characterizing an empirical needle-specific stimulation threshold identified across all treated lumbar and sacral levels.

## Materials and methods

Study design

Retrospective, post-market, open-label, multi-center series. Three independent US surgical sites contributed consecutive patients who underwent MIS lumbar pedicle screw fixation using the ALARA Neuro Access Needle between January 2020 and December 2021. The study was conducted in accordance with the Declaration of Helsinki. Retrospective chart review of de-identified data was reviewed by each site's institutional oversight body. The ALARA Neuro Access Needle Kit holds FDA 510(k) clearance for neuromonitoring applications during pedicle screw cannulation, with a neurostimulation indication granted in 2019.

Patients

Eligible patients were adults (≥18 years) requiring posterior lumbar or lumbosacral stabilization via percutaneous pedicle screws for degenerative disc disease, spinal stenosis, spondylolisthesis, scoliosis, neurogenic claudication, or segmental instability. Patients were excluded if prior surgery at the index level was likely to substantially alter pedicle anatomy, if active spinal infection was present, or if bone quality was radiographically judged inadequate for pedicle fixation at the discretion of the treating surgeon (no formal densitometry threshold was applied). Seventy-five patients met the inclusion criteria (mean age 56.3 years; 25 per surgeon). MIS pedicle screw systems from multiple manufacturers were used at the surgeon's preference; implant systems are not individually reported because this was not a variable of interest. All three surgeons are fellowship-trained orthopedic spine surgeons practicing independently at geographically distinct US institutions: Dr. Mo (spine fellowship, Hospital for Special Surgery, New York; Washington, DC), Dr. Myers (spine fellowship, Cleveland Clinic, Weston, FL; Hollywood, FL), and Dr. Patel (spine fellowship, Los Angeles Spine Institute; Beckley, WV). Their independent participation across three separate institutions, patient populations, and practice settings strengthens the generalizability of the findings.

Device

**Figure 1:**
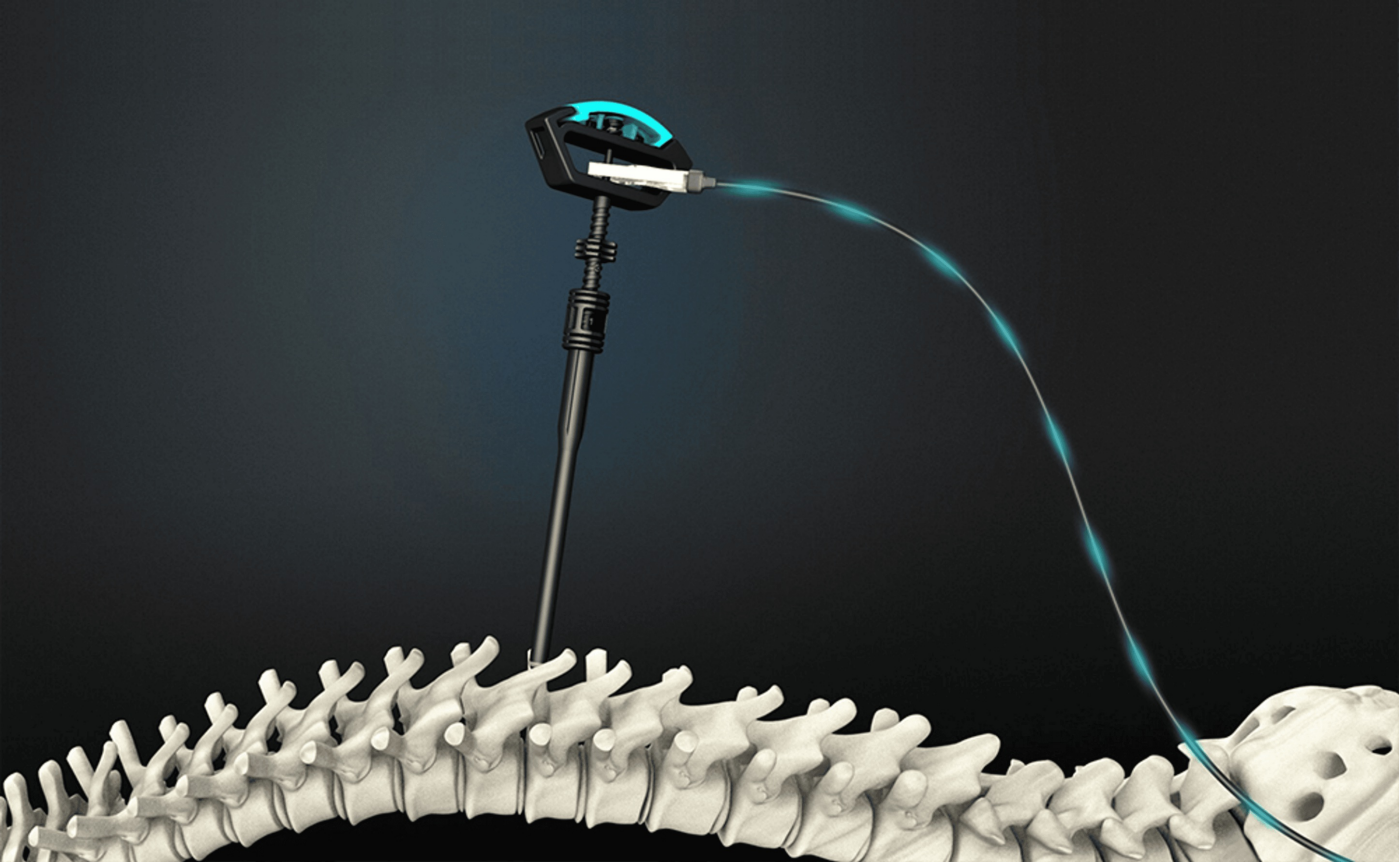
Graphic of ALARA with neuromonitoring connected via neurostimulation clip Image provided by and reproduced with permission from SurGenTec, LLC, the manufacturer and copyright holder of the ALARA™ Access Needle. All rights reserved.

The ALARA Neuro Access Needle Kit is an 11-gauge access needle with an insulated, adjustable, rigid sheath. The sheath is preset by the surgeon to the desired insertion depth and isolates electrical current to the unshielded needle tip. A stainless steel radiopaque ring at the distal end of the sheath provides fluoroscopic depth visualization. The needle is available in bevel-tip or diamond-tip configurations. A neurostimulation clip attaches to the needle handle and delivers continuous electrical stimulation to the existing EMG monitoring system throughout needle advancement (Figure 1). All device-specific claims in this report reflect manufacturer specifications, except where independently observed in the data (stimulation thresholds and repositioning rates).

Procedure

**Figure 2:**
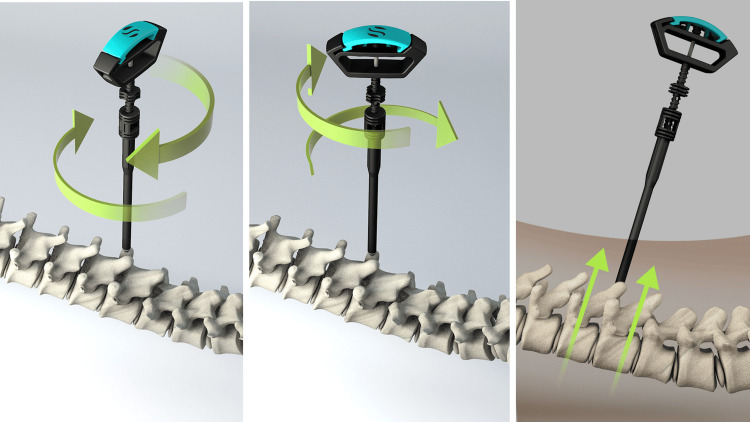
Graphic example of counter-rotation technique for removal of ALARA Image provided by and reproduced with permission from SurGenTec, LLC, the manufacturer and copyright holder of the ALARA™ Access Needle. All rights reserved.

All patients received general anesthesia with total intravenous anesthetic (TIVA) protocols; neuromuscular blockade was avoided from incision through completion of neuromonitoring. Lumbosacral myotomal electrode arrays were placed preoperatively per standard practice. Under biplanar fluoroscopic guidance, the ALARA needle was advanced to the target pedicle. Fluoroscopic confirmation of the radiopaque depth marker against the posterior pedicle cortex preceded initiation of t-EMG stimulation. The neurostimulation clip was connected, and continuous stimulation was maintained during needle advancement. An ALARA needle threshold of ≥5 mA was the minimum accepted value before guidewire placement; this criterion was adopted by surgeon consensus prior to the study period based on clinical experience and was not derived from a pre-specified calibration protocol. Sub-threshold readings prompted fluoroscopic reassessment and needle repositioning. Following acceptable needle threshold confirmation, a guidewire was placed and the needle removed using a counter-rotation technique (Figure 2). Cannulated pedicle screws were placed over the guidewire using each surgeon's standard MIS instrumentation. Final screw position was assessed by biplanar fluoroscopy and t-EMG stimulation. Screws with final readings below 10 mA were repositioned regardless of fluoroscopic appearance.

Outcomes

The primary outcome was the incidence of new postoperative neurological deficit attributable to pedicle screw malposition, assessed by the treating surgeon at discharge and at routine outpatient follow-up. Secondary outcomes included the rate of intraoperative needle repositioning due to sub-threshold readings; the rate of potential pedicle cortical violation, defined as any screw with final t-EMG below 10 mA that could not be corrected to threshold; and characterization of needle-specific EMG threshold values by spinal level (L1-S1). Screw position was assessed by biplanar fluoroscopy and t-EMG only; postoperative CT was not obtained in asymptomatic patients and was therefore not available for anatomical correlation.

Statistics

Descriptive analysis only. Outcomes are presented as counts and proportions. Given the retrospective single-arm design and absence of a control group, no inferential statistical comparisons were made. Threshold values are reported categorically (<15 mA, 15-19 mA, and ≥20 mA), consistent with published benchmarks [7]. This study was not powered for statistical hypothesis testing.

## Results

Patients

Seventy-five patients (mean age 56.3 years; 25 per surgeon) underwent MIS lumbar pedicle screw fixation during the study period. Surgical indications are shown in Table 1. The most common indications were degenerative disc disease and spinal stenosis (22 patients each), followed by spondylolisthesis (17), scoliosis (13), instability (6), and neurogenic claudication (1). Some patients had more than one indication; Table 1 reflects the primary indication recorded at each site. A total of 367 pedicle screws were placed across 182 instrumented spinal levels, reflecting a mean of approximately 2.0 screws per level (bilateral instrumentation at all treated levels except L1, where one patient received a single unilateral screw). Distribution by level is shown in Table 2.

**Table 1:** Primary surgical indications (N=75 patients)¹ ^1^Some patients had co-existing indications; the primary indication as recorded by the treating surgeon is shown.

Primary indication	N (%)
Degenerative disc disease	22 (29.3%)
Spinal stenosis	22 (29.3%)
Spondylolisthesis	17 (22.7%)
Scoliosis	13 (17.3%)
Instability	6 (8.0%)
Neurogenic claudication	1 (1.3%)

**Table 2:** Distribution of instrumented spinal levels² ^2^Number of patients with instrumentation at that level. All levels were instrumented bilaterally (2 screws per level) except L1, where one patient received a single unilateral screw (1 screw). ^3^Total reflects the 75 unique patients in the study. Because most patients underwent instrumentation at more than one spinal level, the per-level patient counts in the "N patients" column sum to more than 75; each patient is counted once at every level treated. Total screws=367. No postoperative CT was performed in asymptomatic patients.

Level	N patients	% of levels	Screws
L1	1	1%	1
L2	11	6%	22
L3	20	11%	40
L4	56	30%	112
L5	61	33%	122
S1	35	19%	70
Total	75^3^	100%	367

Primary outcome: neurological status

No patient sustained a new postoperative neurological deficit attributable to pedicle screw malposition. All 75 patients were assessed by the treating surgeon prior to discharge and at standard outpatient follow-up. This outcome was assessed by clinical neurological examination; no systematic patient-reported outcome instruments were administered. Screw position was assessed by biplanar fluoroscopy and final t-EMG readings only; postoperative CT was not obtained in asymptomatic patients, and anatomical screw position cannot be independently confirmed from these data.

Intraoperative repositioning and potential violation

Of 367 pedicle screws placed, 341 (92.9%) were implanted without needle repositioning. Twenty-six screws (7.1%) required needle repositioning due to sub-threshold ALARA needle EMG readings (<5 mA). In all 26 cases, repositioning was performed before guidewire placement, and final screw readings subsequently achieved acceptable thresholds (≥10 mA). One screw (0.3%) produced persistent sub-threshold EMG readings despite repositioning attempts. This screw was placed at L4 in a patient with degenerative disc disease; the ALARA needle recorded 4 mA, and the final pedicle screw recorded 9 mA. The screw was assessed intraoperatively by fluoroscopy and was interpreted as acceptable by the treating surgeon. The patient was examined postoperatively and demonstrated no neurological deficits at follow-up. These findings are summarized in Table 3.

**Table 3:** Summary of intraoperative EMG outcomes⁵ ^5^Screw position confirmed by biplanar fluoroscopy and t-EMG only. ^6^Defined as final screw t-EMG <10 mA not correctable to threshold; not confirmed by CT. No postoperative CT was performed in asymptomatic patients. EMG: electromyography

Screws placed	Screws repositioned (%)	Potential cortical proximity^6^ (%)	Post-op neurological deficit
367	26 (7.1%)	1 (0.3%)	0 (0%)

Electromyography threshold data by level

**Figure 3:**
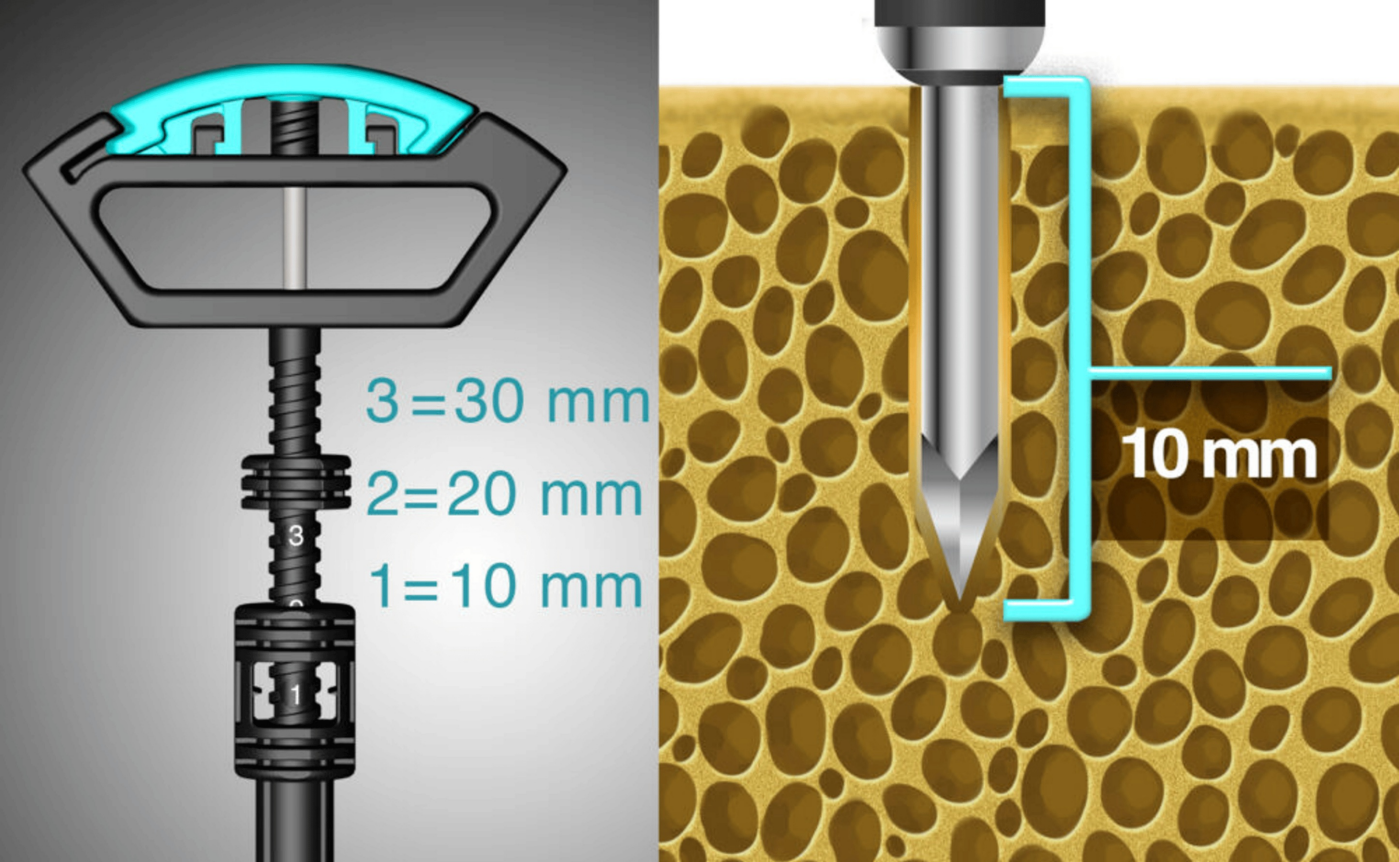
Graphic showing insulation and depth control of ALARA Image provided by and reproduced with permission from SurGenTec, LLC, the manufacturer and copyright holder of the ALARA™ Access Needle. All rights reserved.

ALARA needle threshold readings were characteristically lower than corresponding tap and pedicle screw readings at the same position across all treated levels, consistent with the reduced stimulating surface area of the insulated needle tip (Figure 3) relative to a fully seated screw. Needle readings of ≥5 mA preceded final screw readings of ≥10 mA in all 26 repositioned cases, after repositioning achieved acceptable values, and in all 341 cases placed without repositioning. Among all screws, 72%-75% of final screw readings exceeded 20 mA. Level-by-level EMG distributions are presented in Table 4. No screws at L1 or L2 produced final readings below 15 mA. Sub-threshold needle readings (<5 mA, prompting repositioning) were encountered most frequently at L4 (24 left, 19 right needle readings), L5 (23 left, 23 right), and S1 (20 left, 16 right), consistent with the increased nerve root diameter and more acute pedicle-to-nerve root exit angle at lower lumbar levels, as well as the greater cancellous composition of the S1 pedicle compared with upper lumbar segments [11].

**Table 4:** EMG threshold distribution by spinal level (counts by mA range)⁷ ^7^Values represent the number of readings in each mA range per instrument per side. Needle readings reflect the ALARA Access Needle; these values are instrument-specific and are not directly comparable to screw thresholds. No postoperative CT was performed in asymptomatic patients. L: left; R: right; EMG: electromyography

Level	mA range	L needle (counts)	R needle (counts)	L tap (counts)	R tap (counts)	L screw (counts)	R screw (counts)
L1	≥20	1	1	1	1	1	1
L2	15-19	5	4	1	0	3	1
	≥20	1	3	2	4	3	9
L3	<15	8	9	0	1	1	1
	15-19	3	2	1	0	3	2
	≥20	7	7	5	5	10	16
L4	<15	24	19	1	1	4	4
	15-19	10	18	2	6	6	10
	≥20	19	11	18	13	33	38
L5	<15	23	23	1	0	4	2
	15-19	16	13	3	3	6	10
	≥20	20	24	15	16	40	48
S1	<15	20	16	1	1	2	2
	15-19	5	5	0	2	9	10
	≥20	9	11	7	3	17	20

## Discussion

Principal findings and their limitations

The principal finding of this series is that MIS lumbar pedicle screw fixation using an insulated access needle with continuous t-EMG monitoring was associated with zero clinically evident postoperative neurological deficits across 75 patients and 367 screws at three independent surgical sites. An intraoperative repositioning rate of 7.1% reflects detection of sub-threshold needle readings before guidewire placement, and one screw (0.3%) produced persistent EMG values consistent with possible pedicle cortical proximity despite repositioning. This potential violation was identified by the monitoring system, received additional intraoperative scrutiny, and did not result in neurological sequelae on clinical follow-up.

Screw position was assessed by biplanar fluoroscopy and t-EMG only; no postoperative CT was obtained in asymptomatic patients. What can be stated from these data is that no patient sustained a clinically evident neurological injury and that sub-threshold EMG readings prompted intraoperative correction in 7.1% of cases; the true rate of pedicle cortical breach remains unknown without imaging validation.

**Figure 4:**
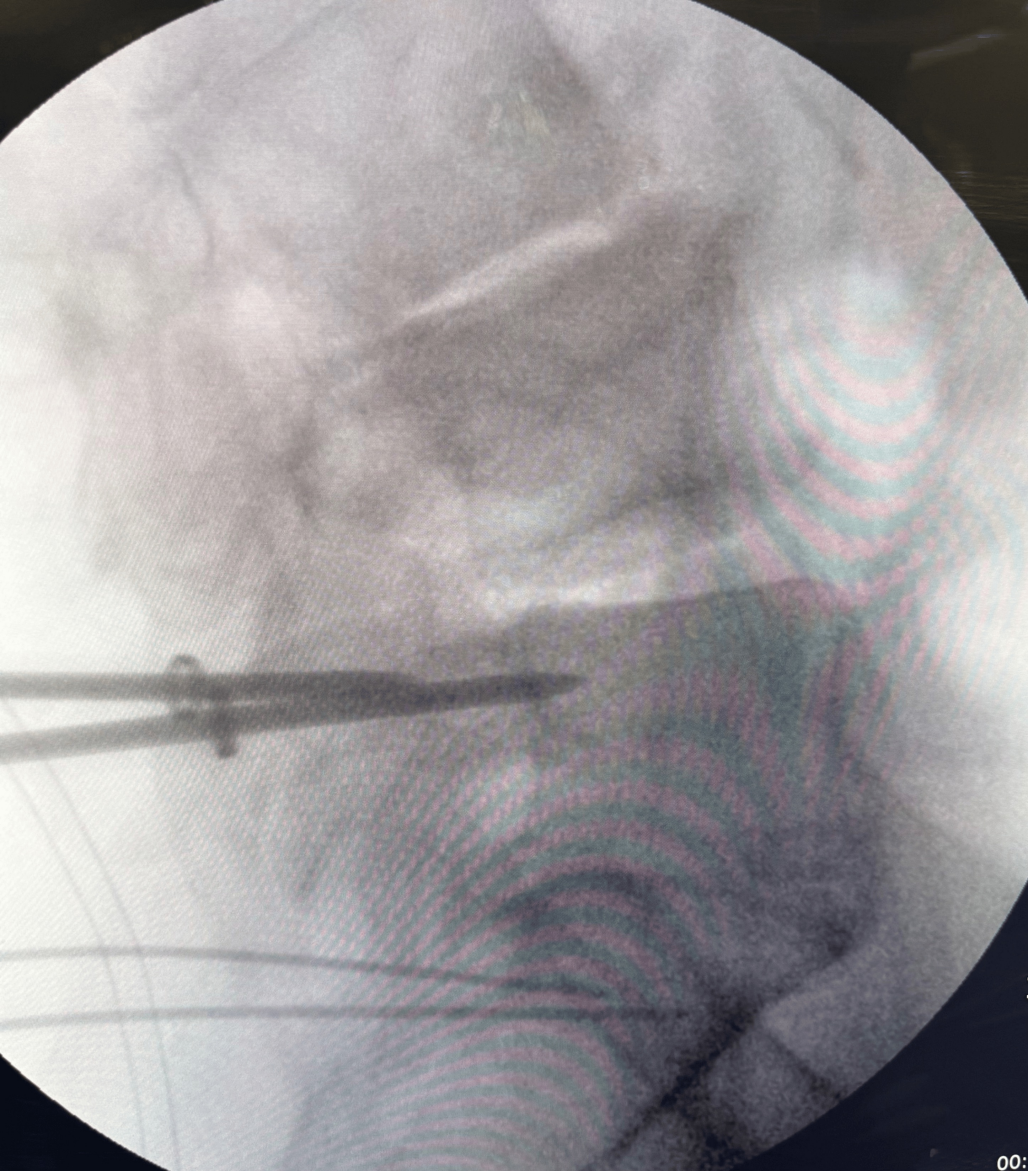
Lateral intraoperative fluoroscopic image showing bilateral ALARA Access Needles placed at L5 during an MIS TLIF Image provided by and reproduced with permission from SurGenTec, LLC, the manufacturer and copyright holder of the ALARA™ Access Needle. All rights reserved. MIS: minimally invasive surgery; TLIF: transforaminal lumbar interbody fusion

**Figure 5:**
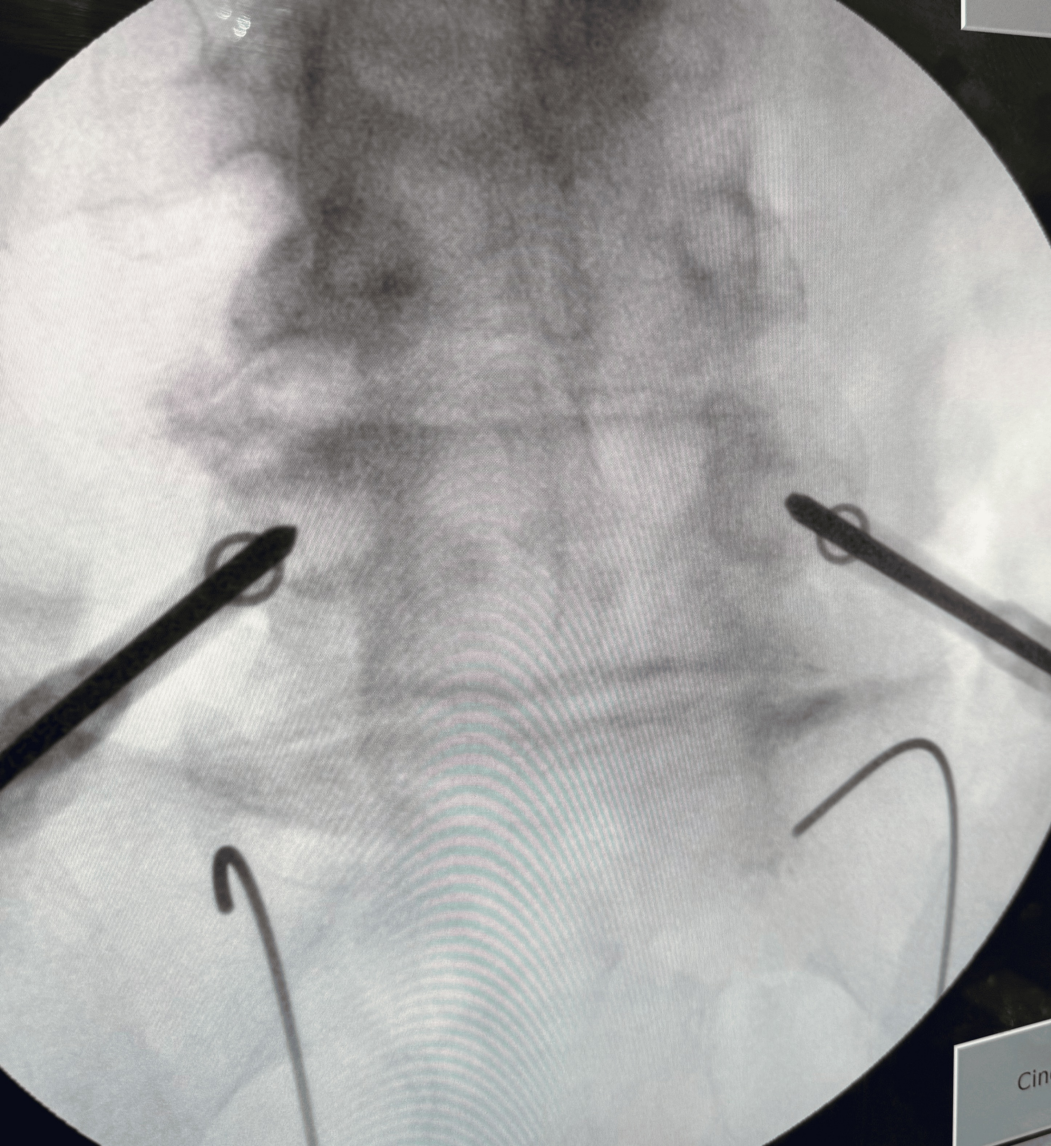
Anteroposterior intraoperative fluoroscopic image demonstrating bilateral ALARA Access Needle placement at L5 Image provided by and reproduced with permission from SurGenTec, LLC, the manufacturer and copyright holder of the ALARA™ Access Needle. All rights reserved.

Representative intraoperative imaging is shown in Figures 4, 5. The images depict bilateral ALARA Access Needles placed at L5 during an MIS transforaminal lumbar interbody fusion (TLIF), with the insulated sheath advanced to the predetermined depth and the radiopaque distal marker confirming sheath apposition to the pedicle prior to t-EMG stimulation. The lateral view (Figure 4) demonstrates the trajectory in the sagittal plane, while the AP view (Figure 5) confirms positioning within the pedicle silhouettes before guidewire placement. The images illustrate the trajectory checkpoint at which biplanar fluoroscopic positioning is paired with t-EMG stimulation; in this case, both criteria were satisfied prior to guidewire placement.

The instrument-specific threshold: why needle and screw values differ

A critical interpretive point for clinicians reading this report is that the ≥5 mA ALARA needle threshold is not equivalent to a 5 mA pedicle screw reading, and the two must not be compared numerically. Published t-EMG danger thresholds, below 10 mA per Glassman et al. [7] and below 8 mA per Lee et al. and Melachuri et al. [9,12], were derived from stimulation applied through pedicle screws or tapped bone channels. These instruments present a large metal-to-bone stimulating interface. EMG response amplitude is governed by current density, defined as current per unit stimulating surface area. A 5 mA threshold at an insulated needle tip reflects a degree of neural proximity comparable to a 15-20 mA threshold at a pedicle screw, as the needle's focused, small-surface electrode delivers current with greater efficiency and precision, while the pedicle screw's larger surface area dissipates current across the surrounding tissue, requiring substantially higher stimulation levels to achieve an equivalent response [8,13].

Across all 367 screws in this series, ALARA needle readings of ≥5 mA preceded final pedicle screw readings of ≥10 mA at the same anatomical position. This approximately two-fold offset was observed consistently across L1 through S1, three surgeons, and multiple implant systems. This consistency is mechanistically coherent; it reflects the proportional increase in stimulating surface area as instrumentation advances from needle tip to fully seated screw, but it has not been validated against postoperative CT, and the precise offset magnitude may vary with needle gauge, bone density, insertion depth, and patient-specific anatomy. Table 5 presents this empirical relationship as a hypothesis-generating framework pending prospective CT-correlated validation; it is not presented as an established equivalence standard.

**Table 5:** Proposed threshold interpretation framework, hypothesis-generating, pending prospective CT validation⁸ ⁸This relationship was empirically observed in this retrospective series and has not been validated against postoperative CT. It should not be applied as an established clinical equivalence standard. Needle and screw values reflect fundamentally different stimulating geometries and cannot be compared numerically. No postoperative CT was performed in asymptomatic patients.

mA reading	Pedicle screw interpretation (published [7-9,12,13])	Insulated needle interpretation (empirical, this series only)	Observed response (this series only)
≥20 mA	High confidence of intrapedicular placement (98% per Glassman et al. [7])	Needle readings well above the observed safety criterion; proceeded without repositioning	Guidewire placed; screws ≥20 mA in 72%-75% of final readings
10-19 mA	Acceptable; screw likely intrapedicular	Above adopted needle criterion (≥5 mA); proceeded	No repositioning; final screw values ≥10 mA
5-9 mA	Danger zone for screws: breach probability 17%-54% at 4-8 mA (Raynor et al. [8])	At or just above the needle criterion. NOT numerically equivalent to a screw reading of 5-9 mA. Empirically observed to precede final screw readings ≥10 mA in this series	At threshold: fluoroscopy confirmed, guidewire placed. Below 5 mA: needle repositioned
<5 mA	Breach probability >54% below 4 mA; 100% below 2.8 mA (Raynor et al. [8])	Below adopted needle criterion. Trajectory unacceptable	Needle repositioned in all 26 cases; acceptable screw thresholds subsequently achieved in 25/26

Repositioning rate in context

Repositioning is the intended clinical response when monitoring detects a suboptimal trajectory; it is not a complication. Kaliya-Perumal et al. reported a 7.8% positive t-EMG response rate among 1856 monitored pedicle screws, with a significant reduction in reoperation rates versus unmonitored surgery [14]. Reddy et al., in a systematic review and meta-analysis, found that patients with a lumbar pedicle screw stimulated below the alarm threshold were ten times more likely to develop a postoperative neurological deficit than those without a low-threshold event [15]. Tong et al., in a CT-confirmed series of 3403 screws, found that t-EMG monitoring reduced medial pedicle breach rates and revision surgery compared with fluoroscopy alone [16]. The 7.1% repositioning rate in this series is consistent with published benchmarks for monitored MIS pedicle screw placement. In the present series, all 26 repositioned screws ultimately achieved acceptable final screw thresholds before definitive implantation, and no patient developed a neurological deficit.

The economic and clinical significance of these 26 needle-stage corrections must be considered in the context of known malposition risk. Population-level data report percutaneous lumbar pedicle screw malposition rates of 3.7% to 31.5%, depending on technique and assessment methodology, a range that underscores how frequently trajectory errors occur even in experienced hands [3]. Revision surgery for symptomatic pedicle screw malposition carries a mean direct cost of approximately $23762 per case (encompassing operating room time, hospital stay, implants, and surgeon reimbursement), with reported US ranges of $17650-$39643 depending on payer mix and case complexity [17]. In high-volume MIS practices, even modest reductions in undetected malposition may therefore translate into meaningful cost avoidance alongside the direct reduction in patient morbidity associated with return-to-OR procedures [18].

Had these 26 suboptimal trajectories proceeded undetected to definitive screw placement, the potential consequences range from nerve root irritation and radiculopathy to permanent motor deficit, outcomes associated not only with significant patient morbidity but also with the medical liability exposure and institutional costs that accompany iatrogenic neurological injury in elective spine surgery [5,17]. Needle-stage detection and correction, by contrast, carry negligible marginal cost and eliminate the trajectory error before any commitment to the screw path is made. The clinical and economic value of this early checkpoint is therefore greatest precisely in the cases where it triggers a correction, the 26 screws in this series, where it did. It should also be noted that neurophysiological monitoring and imaging-based guidance systems address distinct aspects of pedicle screw safety and are generally employed as complementary rather than competing modalities; the present data speak only to the neurophysiological component.

Limitations

This study carries several limitations that must be weighed when interpreting its findings. Postoperative CT was not obtained in asymptomatic patients, meaning screw position was assessed by biplanar fluoroscopy and t-EMG alone; the true rate of pedicle cortical breach cannot be independently confirmed, and the needle-to-screw threshold relationship remains unvalidated against an imaging gold standard. The retrospective, single-arm design without a concurrent non-insulated needle control group prevents isolation of the specific contribution of the insulation property from other device features. While the participation of three fellowship-trained surgeons at independent institutions across geographically distinct US practice settings supports cross-site applicability, the cohort of 75 patients is insufficient for subgroup analyses by bone quality, BMI, or disease etiology. The ≥5 mA needle threshold was established by surgeon consensus prior to the study period rather than derived from a prospective calibration protocol. Prospective studies with mandatory postoperative CT, a matched non-insulated control arm, and formal statistical analysis are needed to establish definitive conclusions.

## Conclusions

In this retrospective multi-center study, continuous needle-stage t-EMG monitoring with the ALARA Access Needle provided real-time trajectory feedback during percutaneous lumbar pedicle screw fixation, allowing the surgeon to identify and correct a suboptimal path before guidewire placement. A central practical finding is that t-EMG thresholds are instrument-specific: a reading taken on the insulated ALARA Access Needle reflects a different stimulating geometry than the same reading on a tap or pedicle screw, and the two cannot be interpreted through a single numerical framework. These findings suggest that needle-stage t-EMG monitoring with the ALARA Access Needle can be applied as a platform-independent procedural adjunct in routine lumbar MIS practice.
